# Left stellate ganglion block, a rescue treatment for ventricular arrhythmia refractory to radiofrequency catheter ablation: A care-compliant case report

**DOI:** 10.1097/MD.0000000000017790

**Published:** 2019-11-01

**Authors:** Shih-Chieh Yang, Chih-Cheng Wu, Yun-Jui Hsieh

**Affiliations:** Taichung Veterans General Hospital, Department of Anesthesiology, 1650 Taiwan Boulevard Sect. 4, Taichung 40705, Taiwan ROC.

**Keywords:** autonomic nerve block, stellate ganglion, ventricular tachycardia

## Abstract

**Rationale:**

Stellate Ganglion Block (SGB) provides a blockade of sympathetic signals from the sympathetic chain and appears to be a promising method of controlling refractory ventricular arrhythmias, but there are scanty data in the literature.

**Patient concerns:**

Herein, we describe a 59-year-old male patient with a history of non-ischemic cardiomyopathy and suffering from frequent VT episodes, who received ICD implantation and regular amiodarone medication control.

**Diagnoses:**

Monomorphic VT refractory to standard medication control and focal extensive catheter ablation.

**Interventions:**

Left Stellate Ganglion Block (LSGB) was performed under ultrasound-assisted injection at the C6 level using a 10 ml solution of 0.4% lidocaine and 0.5% bupivacaine.

**Outcomes:**

In our case, refractory VT subsided and sinus rhythm was retained immediately after LSGB. There were no VT episodes for at least 3 hours during the inter-hospital transfer, which did not involve any specific complications.

**Lessons:**

LSGB may provide effective VT control and play an important role in rescue and bridge therapy before catheter ablation.

## 1 Introduction

Stellate Ganglion Block (SGB) has been widely used in the treatment for various types of acute and chronic pain since it was first introduced in the 1930s.^[1,2]^ It provides a blockade of sympathetic signals from the sympathetic chain. Despite not being part of mainstream treatment, a number of reports have revealed that SGB shows promising effects for controlling refractory ventricular arrhythmias. However, the mechanism by which it exerts its antiarrhythmic effect remains unclear.

We present the case of a patient with refractory ventricular arrhythmia which was controlled by left Stellate Ganglion Block (LSGB). Furthermore, we also review previous studies and discuss the possible pathophysiologies.

## 2 Case report

This 59-year-old male had a history of non-ischemic cardiomyopathy, with chamber dilatation and an ejection fraction (EF) that was 35% as measured two years ago. Cardioversion and Implantable Cardioverter Defibrillator (ICD) implantation, along with amiodarone medication were administered due to frequent Ventricular Tachycardia (VT). Recently, frequent ICD therapy due to fast VT was noted, and the ICD VT detection threshold was adjusted. However, frequent Multifocal VT persisted. Two attempts using focal extensive ablation were performed resulting in limited improvement. The patient also had amiodarone-induced thyrotoxicosis while under Carbimazole treatment.

**Figure 1:**
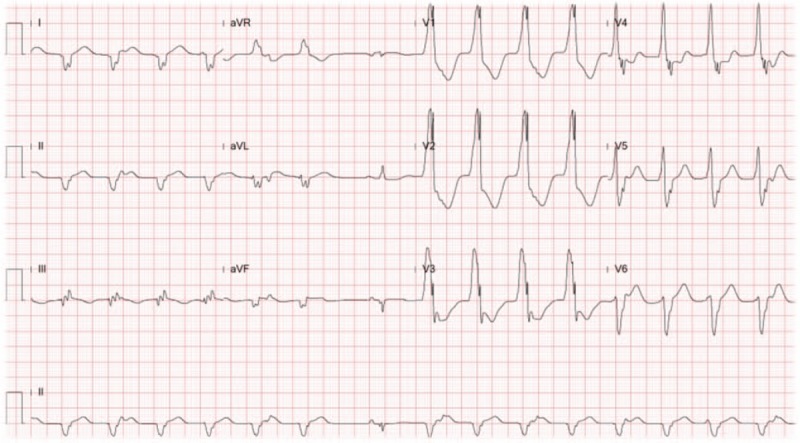
Persistent monomorphic VT recorded by 12-lead ECG.

He was then admitted to hospital due to acute pulmonary edema which improved under diuretic therapy. However, several days later, hemodynamic instability was noted, and an Electrocardiogram (ECG) revealed monomorphic VT (Fig. 1). Emergent intubation and resuscitation were subsequently performed. Lugol's solution, an anti-hyperthyroid agent, and anti-arrhythmics, along with Bisoprolol, Propranolol, Xylocaine and Amiodarone, were administered, and anti-tachycardia pacing with ICD cardioversion were performed, however, VT persisted. Electrical Storm (ES) refractory to medication and defibrillation was suspected, so an interhospital transfer for the purpose of catheter ablation was implemented. Our pain management group was consulted for transient sympathetic blockade.

Informed written consent was obtained from the patient for publication of this case report and accompanying images. Percutaneous LSGB under ultrasound-assisted intervention was then performed. The patient was placed in the supine position with his head tilted to the right side. After preparing a sterile environment, the stellate ganglion was localized and lidocaine was infused locally. Ultrasound-assisted needle insertion to the anterior longus coli fascia plane at the C6 level was performed using a 25-gauge, 1.5-inch needle, implementing the in-plane approach. After the negative aspiration of the syringe showed no air or blood, the LSGB using a 10 ml solution of 0.4% lidocaine and 0.05% bupivacaine was performed smoothly. Sinus rhythm was obtained 10 minutes after infusion. The patient who was in relatively stable condition, was then transferred. No VT episodes occurred during the 3-hour relocation process, and no arrhythmia episodes were observed before catheter ablation.

## 3 Discussion and conclusions

Electrical Storm (ES) refers to a state of cardiac electrical instability, characterized by two or more episodes in a patient without ICD, and three or more episode in a patient with ICD of hemodynamically unstable VT storm, or Ventricular fibrillation (VF) storm within 24 hours.^[3]^ ES is an independent marker for subsequent death among ICD recipients, particularly within the first 3 months of its occurrence (relative risk 5.4).^[4]^ To treat ES, correcting the underlying cause (e.g., acute myocardial infarction, heart failure), and stabilizing the hemodynamic status should be prioritized.

Currently, antiarrhythmic medications and catheter ablation remain the standard treatments for ES.^[5]^ However, there are also other options including neuromodulation with thoracic epidural anesthesia, spinal cord stimulation, cardiac sympathetic denervation, and as in our case, SGB can provide a curative effect in certain situations when standard treatments are ineffective.^[6,7]^

The effect of SGB on heart rhythm was first evaluated in 1966. At the time, animal studies revealed that left stellate stimulation produces prolonged Q-T intervals in open-chested dogs,^[8]^ while LSGB increases the electrophysiological stability of ventricular myocardium in rabbits.^[9]^ These findings imply that the use of LSGB benefit patients with ventricular arrhythmias.

Some case reports have described the effects of SGB on ES. Shrinivas et al treated ES via ultrasound-guided LSGB (0.25% Bupivacaine 6 ml), which resulted in an arrhythmia-free duration of 2 hours immediately after LSGB, and a reduction in the incidence of VT in the following 12 hours.^[10]^ Nademanee et al revealed that in patients with ES treated by sympathetic blockade, 1-week mortality rate (22% vs 82%, *P* < .0001) and 1-year survival rate (67% vs 5%, *P* < .0001) were significantly superior compared with those in patient treated by the ACLS protocol.^[11]^ In a systematic review of 3374 publications, it was found that SGB resulted in a significant decrease in both VA burden (12.4 ± 8.8 vs 1.04 ± 2.12 episodes/day, *P* < .001) and number of external and ICD shocks (10.0 ± 9.1 vs 0.05 ± 0.22 shocks/day, *P* < .01). Following SGB, 80.6% of patients survived through to discharge.^[12]^

In our case, it was necessary to conduct an inter-hospital transfer before catheter ablation, and therefore it was especially important to perform rescue therapy for VT refractory to medication control. As such, we successfully performed LSGB and sinus rhythm was retained immediately. There were no VT episodes for at least 3 hours during the inter-hospital transfer which did not involve any complications.

In conclusion, although it is not the mainstay treatment, LSGB may provide effective arrhythmia control and play an important role in rescue and bridge therapy, especially for the treatment of refractory ventricular arrhythmia before catheter ablation.

## Acknowledgments

We received no funding from any organizations or groups, and we declare no conflicts of interest in this or other relevant studies.

## Author contributions

**Conceptualization:** Yun-Jui Hsieh.

**Investigation:** Yun-Jui Hsieh.

**Writing – original draft:** Yun-Jui Hsieh, Shih-Chieh Yang, Chih-Cheng Wu.

**Writing – review & editing:** Yun-Jui Hsieh, Shih-Chieh Yang, Chih-Cheng Wu.

Yun-Jui Hsieh orcid: 0000-0003-1012-7227.

## References

[1] Kulkarni KR Kadam AI Namazi IJ Efficacy of stellate ganglion block with an adjuvant ketamine for peripheral vascular disease of the upper limbs. Indian J Anaesth 2010;54:546–51. doi:10.4103/0019-5049.72645. 21224973 10.4103/0019-5049.72645 PMC3016576

[2] Serna-Gutiérrez J Bloqueo ganglio estrellado guiado por ultrasonografia. Rev Colomb Anestesiol 2015;43:278–82. doi:10.1016/j.rcae.2014.09.004.

[3] Kowey PR An overview of antiarrhythmic drug management of electrical storm. Can J Cardiol 1996;12 Suppl B:3B. 8616726

[4] Exner DV Pinski SL Wyse DG Electrical storm presages nonsudden death: The antiarrhythmics versus implantable defibrillators (AVID) trial. Circulation 2001;103:2066–71. 11319196 10.1161/01.cir.103.16.2066

[5] Zipes DP Camm AJ Borggrefe M ACC/AHA/ESC 2006 guidelines for management of patients with ventricular arrhythmias and the prevention of sudden cardiac death–executive summary: A report of the American College of Cardiology/American Heart Association Task Force and the European Society of Cardiology Committee for Practice Guidelines (Writing Committee to Develop Guidelines for Management of Patients with Ventricular Arrhythmias and the Prevention of Sudden Cardiac Death) Developed in collaboration with the European Heart Rhythm Association and the Heart Rhythm Society. J Am Coll Cardiol 2006;48:1064–108. 10.1016/j.jacc.2006.07.010 16949478

[6] Blomberg S Ricksten SE Thoracic epidural anaesthesia decreases the incidence of ventricular arrhythmias during acute myocardial ischaemia in the anaesthetized rat. Acta Anaesthesiol Scand 1988;32:173–8. 3364144 10.1111/j.1399-6576.1988.tb02710.x

[7] Kamibayashi T Hayashi Y Mammoto T Thoracic epidural anesthesia attenuates halothane-induced myocardial sensitization to dysrhythmogenic effect of epinephrine in dogs. Anesthesiology 1995;82:129–34. 7832294 10.1097/00000542-199501000-00017

[8] Yanowitz F Preston JB Abildskov JA Functional distribution of right and left stellate innervation to the ventricles. Production of neurogenic electrocardiographic changes by unilateral alteration of sympathetic tone. Circ Res 1966;18:416–28. 4952701 10.1161/01.res.18.4.416

[9] Gu Y Wang L Wang X Assessment of ventricular electrophysiological characteristics at periinfarct zone of postmyocardial infarction in rabbits following stellate ganglion block. J Cardiovasc Electrophysiol 2012;23:S29–35. 10.1111/j.1540-8167.2012.02437.x 22994966

[10] Shrinivas G Rupa S Unnikrishnan M Electrical storm: Role of stellate ganglion blockade and anesthetic implications of left cardiac sympathetic denervation. Indian J Anaesth 2013;57:397–400. 24163457 10.4103/0019-5049.118568 PMC3800335

[11] Nademanee K Taylor R Bailey WE Treating electrical storm: Sympathetic blockade versus advanced cardiac life support-guided therapy. Circulation 2000;102:742–7. 10942741 10.1161/01.cir.102.7.742

[12] Meng L Tseng CH Shivkumar K Efficacy of stellate ganglion blockade in managing electrical storm: a systematic review. JACC Clin Electrophysiol 2017;3:942–9. doi: 10.1016/j.jacep.2017.06.006. 29270467 10.1016/j.jacep.2017.06.006 PMC5734652

